# Telemetry data of red king crab (*Paralithodes camtschaticus*) migrations in a north Norwegian fjord

**DOI:** 10.1016/j.dib.2022.107894

**Published:** 2022-02-02

**Authors:** Magnus Aune, Jenny L.A. Jensen, Guttorm N. Christensen, Kåre Tormod Nilsen, Benjamin Merkel, Paul E. Renaud

**Affiliations:** a Akvaplan-niva AS, Fram Centre, 9007 Tromsø, Norway; b K-To-9 AS, Fuglenesveien 101, 9601 Hammerfest, Norway; c University Centre in Svalbard, 9071 Longyearbyen, Norway

**Keywords:** Acoustic telemetry, Crustaceans, Movement, Oceanography, Tracking

## Abstract

Acoustic telemetry allows for high-resolution, long-term tracking of moving animals. Here, we describe data on the movement patterns of 37 adult red king crab (RKC, *Paralithodes camtschaticus*) obtained by means of acoustic telemetry. Acoustically tagged RKC were released in Gamvikfjorden (Sørøya, northern Norway) the 24th of May 2016 and tracked until the 1st of November 2016. Individual crabs resided in the fjord for 1–162 days and were recorded 16 - 11,501 times (mean number of records per crab: 2,851). In total, the data set consist of 105,484 pairs of accurate spatio-temporal coordinates. The acoustic receivers (*n* = 38) deployed close to the seabed were integrated with temperature sensors that continuously recorded the ambient seawater temperature, resulting in 174,154 water temperature recordings. These novel tracking data can be used to investigate the species' migratory behaviour, spatio-temporal habitat selection, and the relative role of their environment and their possible food sources. Moreover, the high-resolution seawater temperature dataset may serve independently as input data in physical-oceanographic models of this sub-Arctic sill fjord.

## Specifications Table

**Table:** 

Subject	Ecology and behaviour
Specific subject area	Migratory behaviour
Type of data	Tables Animation
How data were acquired	Data were acquired using acoustic telemetry, tracking red king crab movements and recording water temperature over a 162-day period.
Data format	Triangulated raw data Raw data
Parameters for data collection	Sex, body size (carapace length and width) and weight of individual crabs. Spatial coordinates and associated time indications describing movements of individual crabs. Seawater temperature (°C).
Description of data collection	Acoustic receivers were deployed close to the seabed in a regular grid throughout the study area. Acoustic tags were attached to adult individuals of red king crabs. The movements of the crabs within the study area were tracked over a 5-month period (from the 24th of May until the 1st of November 2016). Temperature sensors attached to the acoustic receivers recorded the seawater temperature continuously.
Data source location	Data was collected in Gamvikfjorden, a fjord located on the north-western part of the island Sørøya, northern Norway (70° 47′ N, 23° 16′ E)
Data accessibility	Repository name: [Zenodo] Data identification number: [10.5281/zenodo.5823340] Direct URL to data: [https://doi.org/10.5281/zenodo.5823339]
Related research article	M. Aune, J.L. Jensen, S.I. Siikavuopio, G.N. Christensen, K.T Nilsen, B. Merkel, P.E. Renaud. Space and habitat utilization of the red king crab (*Paralithodes camtschaticus*) in a newly invaded fjord in northern Norway. Frontiers in Marine Science, in press [3]

## Value of the Data

• High-resolution data and knowledge of migration patterns and habitat utilization is required in order to facilitate rigorous management and efficient harvesting of the abundant, migratory and commercially attractive red king crab.
• These data will benefit managers, scientists, the fishing industry and hobby fishermen.
• Researchers can use the datasets to develop habitat selection models for this species and test the relative importance of available food sources and environmental constraints.

## 1 Data Description

Data obtained by 38 acoustic receivers (Table 1) resulted in a position dataset consisting of 105,484 pairs of accurate spatial coordinates and associated time indications, describing the seasonal movements of the 37 tagged red king crabs (RKC) within the study area (summarized in Table 2). In addition, the temperature sensors attached to the acoustic receivers generated 174,154 water temperature recordings. An animation covering the period from the 1st of June until the 1st of September 2016 provides an overview of the RKC movement data (Supplementary Files 1A and 1B). The depth of each acoustic receiver is provided (Table 1). Also, the sex, body weight, carapace length and carapace width of each crab was registered (Table 2). Based on the dates of release and last registration, the residence time of each crab within the study area was calculated. Some crabs left the study area a few hours or days after release, and were consequently registered relatively few times (typically <200 times). On the other hand, other crabs resided in the fjord throughout the study period (262 days) and were typically registered several thousand times (Table 2).

**Table 1:** Positions of 38 acoustic receivers applied in Gamvikfjorden (Sørøya, northern Norway) to track individuals of the red king crab (*Paralithodes camtschaticus*) in the period 24th of May until the 1st of November 2016. Depth is given in meters. "Synchronization tag?" indicates whether a receiver was equipped with a synchronization tag.

Receiver ID	Latitude (°N)	Longitude (°E)	Depth (m)	Synchronization tag?
96	70.77239	23.28890	18	No
97	70.77502	23.27484	41	No
98	70.77659	23.28654	40	No
99	70.77916	23.26669	33	No
100	70.77850	23.27750	61	Yes
102	70.78451	23.26340	21	No
106	70.78819	23.27021	58	Yes
107	70.79092	23.28007	34	No
108	70.79319	23.25090	24	Yes
109	70.79279	23.26540	53	No
110	70.79564	23.27854	43	Yes
111	70.79166	23.29560	15	No
113	70.79679	23.23861	20	No
114	70.79710	23.25648	53	No
116	70.80059	23.28368	32	No
117	70.79597	23.29308	25	No
118	70.79932	23.29592	15	No
119	70.79514	23.30916	15	Yes
120	70.79022	23.30830	18	No
121	70.79156	23.32201	9	No
122	70.80066	23.23269	17	No
123	70.80155	23.24809	54	Yes
124	70.80234	23.26207	46	No
126	70.80479	23.29106	15	No
127	70.80454	23.22498	15	No
128	70.80494	23.23842	51	No
130	70.80696	23.26917	32	No
131	70.80767	23.28214	27	No
133	70.80991	23.22168	26	No
134	70.80908	23.23296	48	Yes
135	70.80949	23.24538	42	No
137	70.81165	23.27450	36	No
138	70.81308	23.28618	26	Yes
140	70.81463	23.29688	20	No
156	70.81378	23.23834	32	No
157	70.81395	23.25155	42	No
158	70.81503	23.26493	39	No
163	70.81366	23.22638	50	No

**Table 2:** Overview of biological properties of acoustically tagged RKC, and data of their residence time in Gamvikfjorden, northern Norway, in 2016.

Crab ID	Sex	Body weight (kg)	Carapace length (cm)	Carapace width (cm)	Release date	Date of last registration	Residence time (days)	Number of registrations
R64K-749	Male	2.6	18.5	18	24.05.2016	24.05.2016	1	16
S256–239	Female	1.9	18	16.5	24.05.2016	24.05.2016	1	25
S256–241	Female	1.6	17	15	24.05.2016	24.05.2016	1	17
R64K-748	Male	3	18.5	20	24.05.2016	25.05.2016	2	52
R64K-766	Male	1.6	15.5	15	24.05.2016	25.05.2016	2	32
S256–236	Male	2.9	18	18	24.05.2016	25.05.2016	2	47
S256–237	Male	2.2	17.5	17	24.05.2016	25.05.2016	2	16
S256–238	Male	1.3	16	14	24.05.2016	25.05.2016	2	29
R64K-750	Male	2.1	17	17	24.05.2016	26.05.2016	3	151
R64K-762	Male	2.9	19.5	19	24.05.2016	26.05.2016	3	86
R64K-768	Female	1.5	16	14.5	24.05.2016	26.05.2016	3	117
S256–234	Male	2.9	19	20	24.05.2016	26.05.2016	3	112
R64K-754	Female	1.2	16	13	24.05.2016	30.05.2016	7	389
S256–235	Male	3.2	20.5	19.5	24.05.2016	01.06.2016	9	132
S256–243	Female	1.6	16.5	14.5	24.05.2016	02.06.2016	10	1083
R64K-759	Male	2.9	19	18	24.05.2016	03.06.2016	11	230
R64K-765	Male	1.7	16	15.5	24.05.2016	13.07.2016	51	778
S256–240	Female	1.9	18	15	24.05.2016	04.08.2016	73	1112
S256–233	Male	2.6	17	17.5	24.05.2016	28.08.2016	97	5368
S256–242	Female	1.7	18	15.5	24.05.2016	21.09.2016	121	3945
R64K-756	Female	1.3	16	14	24.05.2016	04.10.2016	134	2092
R64K-746	Male	1.9	16	15.5	24.05.2016	26.10.2016	156	1869
R64K-757	Female	1.4	16	15	24.05.2016	30.10.2016	160	10,489
R64K-758	Male	1.7	17	16	24.05.2016	30.10.2016	160	2026
S256–231	Male	2.7	20	19	24.05.2016	30.10.2016	160	4633
R64K-747	Male	3.4	19.5	20	24.05.2016	01.11.2016	162	5034
R64K-751	Female	1.7	18.5	14.5	24.05.2016	01.11.2016	162	3643
R64K-752	Female	1.3	16	13	24.05.2016	01.11.2016	162	4430
R64K-753	Female	1.3	16	14	24.05.2016	01.11.2016	162	4784
R64K-760	Male	3	19.5	19.5	24.05.2016	01.11.2016	162	9751
R64K-761	Male	3.5	20	20	24.05.2016	01.11.2016	162	9828
R64K-763	Male	2.6	17	17.5	24.05.2016	01.11.2016	162	1624
R64K-764	Male	2.3	17	17.5	24.05.2016	01.11.2016	162	11,501
R64K-767	Male	1.9	15.5	15	24.05.2016	01.11.2016	162	2678
S256–232	Male	2.7	19	18.5	24.05.2016	01.11.2016	162	1058
S256–244	Female	1.4	18	14	24.05.2016	01.11.2016	162	6081
S256–245	Male	1.8	16	15	24.05.2016	01.11.2016	162	10,226

## 2 Experimental Design, Materials and Methods

### 2.1 Study area

**Fig. 1:**
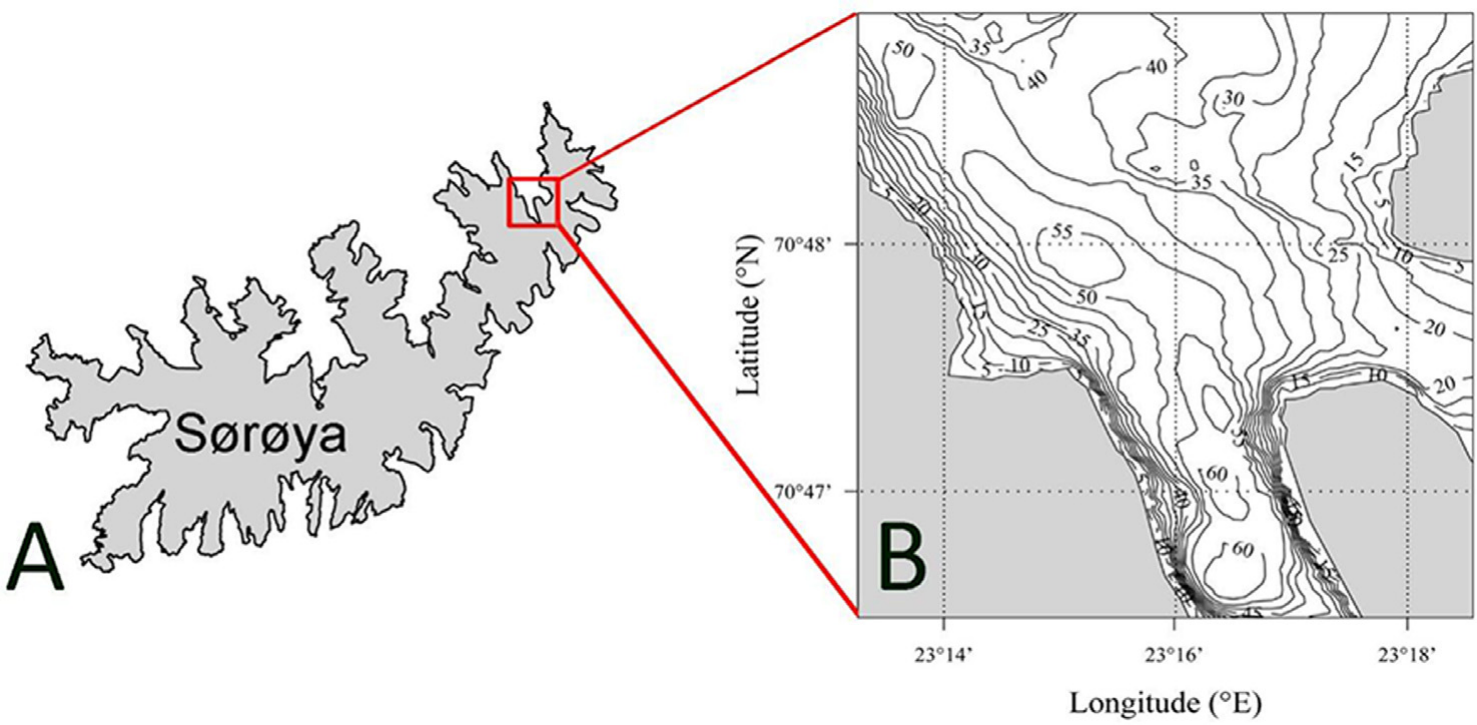
Maps of the study area. A) The island Sørøya, located in northern Norway. B) Gamvikfjorden, a fjord located on the north-western side of Sørøya.

Acoustic telemetry data on the red king crab were collected in Gamvikfjorden, a fjord located on the north-western side of the island Sørøya, northern Norway (Fig. 1). The study area covered an area of ∼7 km^2^, and the maximum water depth was ∼62 m. The bathymetry of the fjord is characterized by steep slopes on the western side and in the inner parts, flat and sandy areas on the eastern side, and a deep trench along the centre of the fjord.

### 2.2 Tagging, tracking, and positioning

By use of baited square pots, 37 adult individuals of RKC (24 males and 13 females) were caught in Gamvikfjorden on the 24th of May 2016. The crabs were brought on board the research vessel, and an acoustic transmitter was attached to the merus of the fourth pereopod by use of cable ties. A small plastic note with contact information for recaptures were also attached to the tag. The crabs were tagged with either acoustic identification transmitters (*n* = 22, tag type ATID-MP-13, transmission rate: 240–360 s random interval, diameter: 133 mm, length: 17 mm, mass in air/water: 1.8/1.1 g, lifespan: 36 months, Thelma Biotel AS, Norway; www.thelmabiotel.com) or depth transmitters (*n* = 15, tag type ADT-MP-13, transmission rate: 240–360 s random interval, diameter: 13 mm, length: 21.5 mm, weight in air/water: 2.0/1.1 g, lifespan: 36 months, Thelma Biotel AS). The body weight (BW) ranged from 1.2 to 3.5 kg (mean mass 2.5 kg ± 0.6 kg for males; and 1.5 kg ± 0.2 kg for females), carapace length (CL) ranged from 15.5 to 20.5 cm (mean length 17.9 cm ± 1.6 cm for males and 16.9 cm ± 1.0 cm for females), and carapace width (CW) ranged from 13 to 20 cm (mean 17.6 cm ± 1.9 cm for males and 14.5 cm ± 1.0 cm for females) (Table 1). The RKC attains sexual maturation at a CL of ∼110 mm [1], and we therefore assume that all individuals were mature. After tagging, the crabs were released back into the water at the same location where they were captured.

In total, 54 acoustic receivers equipped with temperature sensors (TBR-700, Thelma Biotel AS) were deployed in Gamvikfjorden on the 25th of April until the 1st of November 2016 in order to track the movements of the tagged crabs (Table 2). The precision of the temperature sensors was ±0.1 °C. The receivers were attached to a rope on an anchored buoy and suspended with the antennae facing down 5 m above the sea floor. An extra floater was attached to the rope 1 m above the receiver, to insure a vertical position of the receiver at low tides. The receivers were distributed in the study area according to a grid system that allows triangulation of accurate positions within the study area (as described in Davidsen et al. 2019 [2], positioning performed by Thelma Biotel AS), and 10 synchronisation tags (tag type ART-HP-16, transmission rate: 540–660 s random interval, lifespan: 36 months, Thelma Biotel AS) were used for correction of receiver clocks. Rough weather conditions caused the loss of 16 receivers (many of which were later recovered on nearby beaches), which induced some areas of limited acoustic coverage, particularly in the southern part of the study area.

## Ethics Statement

The authors confirm that all experiments comply with the ARRIVE guidelines and were carried out in accordance with the U.K. Animals (Scientific Procedures) Act, 1986 and associated guidelines, EU Directive 2010/63/EU for animal experiments, or the National Institutes of Health guide for the care and use of Laboratory animals (NIH Publications No. 8023, revised 1978).

## CRediT authorship contribution statement

**Magnus Aune:** Writing – original draft, Conceptualization, Data curation. **Jenny L.A. Jensen:** Data curation, Conceptualization, Methodology, Writing – review & editing. **Guttorm N. Christensen:** Conceptualization, Methodology, Writing – review & editing. **Kåre Tormod Nilsen:** Conceptualization, Writing – review & editing. **Benjamin Merkel:** Writing – review & editing. **Paul E. Renaud:** Writing – review & editing.

## Declaration of Competing Interest

The authors declare that they have no known competing financial interests or personal relationships which have or could be perceived to have influenced the work reported in this article.

## Appendix Supplementary materials

Image, image 1
