# Supplementary figures and images for: Telemetry data of red king crab (Paralithodes camtschaticus) migrations in a north Norwegian fjord
Source: Data Brief. 2022 Feb 2;41:107894. doi: 10.1016/j.dib.2022.107894 (PMC8844838; doi:10.1016/j.dib.2022.107894)

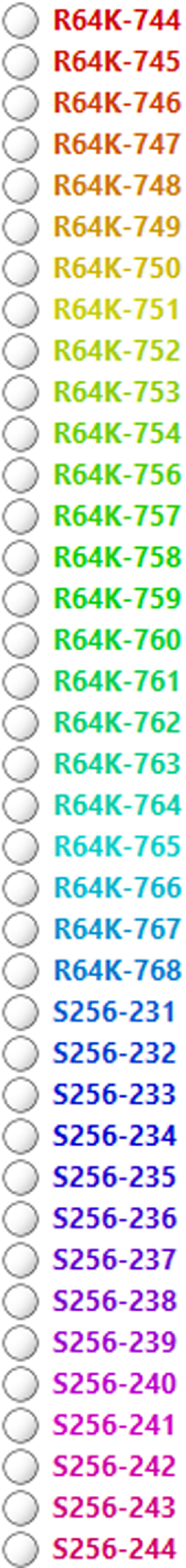

Supplement: Supplementary file 1 [file mmc1.jpg]
